# Pathogenic Glomulin Gene Variant in a Patient with Idiopathic Pulmonary Arterial Hypertension: A Novel Association Case Report

**DOI:** 10.3390/reports8040209

**Published:** 2025-10-20

**Authors:** Ilias E. Dimeas, George E. Dimeas, George E. Zakynthinos, Cormac McCarthy, Zoe Daniil, Georgia Xiromerisiou

**Affiliations:** 1 Department of Respiratory Medicine, University Hospital of Larissa, 41110 Larissa, Greece; gedim06@hotmail.com (G.E.D.); zdaniil@uth.gr (Z.D.); 2 School of Medicine, University College Dublin, D04 V1W8 Dublin, Ireland; cormac.mccarthy@ucd.ie; 3 Department of Respiratory Medicine, St. Vincent’s University Hospital, D04 T6F4 Dublin, Ireland; 4 Department of Internal Medicine, General Hospital of Karditsa, 43100 Karditsa, Greece; 5 3rd Department of Cardiology, “Sotiria” Chest Diseases Hospital, Medical School, National and Kapodistrian University of Athens, 11527 Athens, Greece; gzakynthinos2@gmail.com; 6 Department of Neurology, Faculty of Medicine, University of Thessaly, 41500 Larissa, Greece; georgiaxiromerisiou@gmail.com

**Keywords:** pulmonary arterial hypertension, glomulin (GLMN) gene, vascular smooth muscle, whole-genome sequencing, respiratory muscle weakness, case report

## Abstract

**Background and Clinical Significance:** Idiopathic pulmonary arterial hypertension is a rare disorder, often linked to genetic predisposition. Canonical pulmonary arterial hypertension genes such as BMPR2, KCNK3, and TBX4 are well described, but novel associations continue to emerge. Glomulin (GLMN) encodes a protein essential for vascular smooth-muscle biology, classically implicated in glomuvenous malformations, yet not previously associated with pulmonary arterial hypertension. **Case Presentation:** We present a 49-year-old woman with progressive dyspnea, edema, and persistent hypercapnic respiratory failure. Right-heart catheterization confirmed precapillary pulmonary hypertension. Comprehensive evaluation, including ventilation/perfusion scanning, autoimmune panel, polysomnography, and high-resolution computed tomography, excluded secondary causes. Respiratory assessment revealed diaphragmatic weakness and reduced respiratory muscle pressures, consistent with primary myopathy and explaining the unusual hypercapnic profile. Whole-genome sequencing identified a heterozygous pathogenic GLMN nonsense variant, while canonical pulmonary arterial hypertension genes were negative. No cutaneous or mucosal glomuvenous malformations were found. The patient was treated with oxygen therapy, diuretics, non-invasive ventilation, and dual oral pulmonary arterial hypertension therapy (ambrisentan and tadalafil), with stabilization but persistent hypercapnia. **Conclusions:** To our knowledge, this is the first reported co-occurrence of idiopathic pulmonary arterial hypertension and a pathogenic GLMN variant. While causality cannot be inferred, glomulin’s role in vascular smooth-muscle maturation provides a plausible link to pulmonary vascular remodeling. This case underscores the importance of assessing respiratory muscle function in idiopathic pulmonary arterial hypertension patients with hypercapnia and highlights the potential relevance of extended genetic testing in rare pulmonary vascular disease.

## 1. Introduction and Clinical Significance

Idiopathic pulmonary arterial hypertension (IPAH) is a rare, progressive disorder characterized by pulmonary vascular remodeling and increased pulmonary vascular resistance, leading to right ventricular failure and premature death [1]. According to the 2022 European Society of Cardiology/European Respiratory Society (ESC/ERS) guidelines, IPAH is defined hemodynamically by a mean pulmonary artery pressure (mPAP) > 20 mmHg, pulmonary arterial wedge pressure (PAWP) ≤ 15 mmHg, and pulmonary vascular resistance (PVR) > 2 Wood units [1]. Patients typically present with exertional dyspnea, fatigue, and right-sided heart failure. In arterial blood gases, mild hypoxemia and hypocapnia are common, reflecting hyperventilation rather than ventilatory pump failure [2].

Genetic predisposition plays a crucial role in IPAH pathogenesis. Mutations in BMPR2 account for most heritable cases, while variants in KCNK3, TBX4, CAV1, SOX17, ATP13A3, and EIF2AK4 have also been implicated [3]. Genetic testing is therefore recommended in patients with idiopathic or familial disease [1,3]. Glomulin (GLMN) encodes a protein essential for vascular smooth muscle cell development and stabilization of vascular structures [4]. Pathogenic GLMN variants are classically associated with glomuvenous malformations (GVMs), cutaneous or mucosal venous lesions with incomplete penetrance and variable expressivity [4]. To date, pulmonary vascular disease has not been linked to GLMN variants.

We report a patient with IPAH complicated by persistent hypercapnic respiratory failure, attributable to primary myopathy with diaphragmatic weakness. Genetic analysis identified a pathogenic GLMN nonsense variant, while canonical pulmonary arterial hypertension (PAH) genes were negative. Although causality cannot be established, glomulin’s role in vascular smooth muscle biology may suggest a plausible contribution to pulmonary vascular remodeling. Loss of glomulin function may impair vascular smooth-muscle maturation and stability, thereby favoring maladaptive pulmonary vascular remodeling. To our knowledge, this represents the first reported co-occurrence of IPAH and a pathogenic GLMN variant.

## 2. Case Presentation

A Greek 49-year-old woman was admitted with progressive exertional dyspnea, bilateral lower-limb oedema, and fatigue. She had no history of smoking, illicit drug use, or exposure to agents associated with PAH. There was no family history of pulmonary hypertension (PH), sudden cardiac death, or neuromuscular disease. Her past medical history included afebrile episodes of dyspnea, and cough attributed to respiratory infections, but she had no connective tissue disease, autoimmune manifestations, or systemic comorbidities.

On admission, she was afebrile and hemodynamically stable. Physical examination revealed jugular venous distension, right ventricular heave, bilateral ankle oedema, and reduced diaphragmatic excursion. Neurological examination revealed reduced proximal muscle strength in both upper and lower limbs. Lung auscultation revealed diminished breath sounds at the bases, without adventitious sounds. Arterial blood gases (ABG) on an inspired oxygen fraction (FiO_2_) of 0.21 showed pH 7.37 (normal 7.35–7.45), partial pressure of carbon dioxide (PaCO_2_) 72 mmHg (normal 35–45 mmHg), partial pressure of oxygen (PaO_2_) 58 mmHg (normal 80–100 mmHg), and bicarbonate (HCO_3_^−^) 40 mmol/L (normal 22–28 mmol/L), indicating chronic compensated hypercapnic respiratory failure. Routine laboratory tests, including complete blood count, liver function tests and renal function were within normal limits.

**Figure 1:**
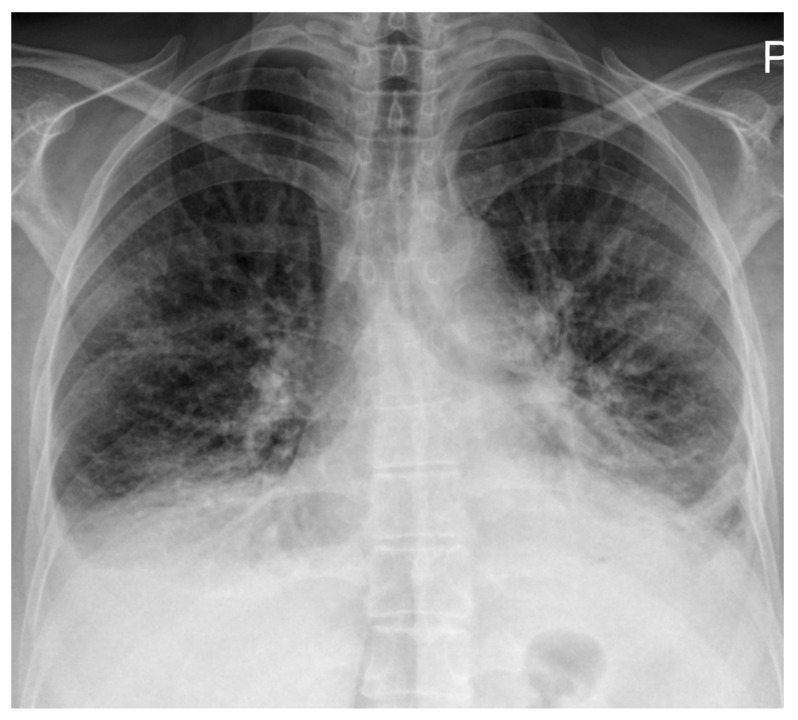
Chest radiography on admission demonstrating cardiomegaly with dilation of the central pulmonary arteries, increased interstitial markings with a perihilar “bat-wing” pattern, and bilateral pleural effusions, all indicative of pulmonary congestion. Bilateral elevation of the hemidiaphragms is also noted, possibly related to underlying myopathy.

Electrocardiography demonstrated right atrial enlargement and right ventricular strain. Chest radiography (Figure 1) revealed cardiomegaly, pulmonary congestion with enlarged central pulmonary arteries, bilateral pleural effusions, and elevation of both hemidiaphragms.

**Figure 2:**
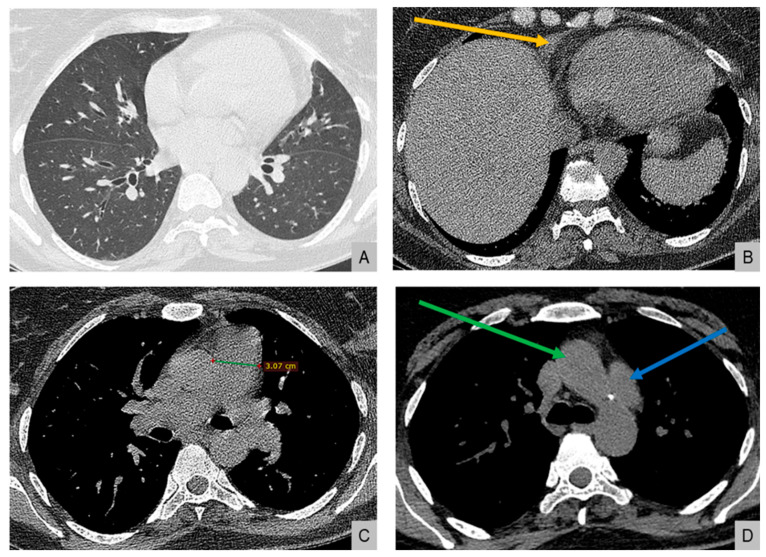
High-resolution computed tomography. (**A**) Normal lung parenchyma. (**B**) Small pericardial effusion (orange arrow). (**C**) Dilated main pulmonary artery measuring 30.7 mm (normal <27 mm in females). (**D**) The “egg-and-banana sign” in pulmonary hypertension: the dilated pulmonary artery (blue arrow, ‘egg’) seen above the lower margin of the aortic arch (green arrow, ‘banana’).

High-resolution computed tomography (HRCT) (Figure 2) showed markedly dilated pulmonary arteries, a small pericardial effusion, and absence of interstitial lung disease or features of pulmonary veno-occlusive disease. The “egg-and-banana sign” was observed (Figure 2D), with the main pulmonary artery appearing enlarged and ovoid (‘egg’) above the aortic arch (‘banana’), a pattern typically seen in pulmonary hypertension.

Echocardiography estimated a pulmonary artery systolic pressure of 80–90 mmHg, with severe right atrial and right ventricular dilatation and preserved left ventricular systolic function. Ventilation/perfusion scanning demonstrated no mismatched perfusion defects, excluding chronic thromboembolic pulmonary hypertension. Right-to-left shunt testing was negative.

Pulmonary function testing showed preserved spirometry with forced expiratory volume in one second (FEV_1_) 78% predicted, forced vital capacity (FVC) 70% predicted, and an FEV_1_/FVC ratio of 0.89. Total lung capacity (TLC) was mildly reduced at 72% predicted, consistent with a restrictive pattern likely due to impaired diaphragmatic excursion. Diffusing capacity for carbon monoxide (DLCO) was reduced at 38% of the predicted. N-terminal pro–B-type natriuretic peptide (NT-proBNP) was elevated at 2600 pg/mL (normal <125 pg/mL). The patient was in World Health Organization functional class (WHO-FC) III at baseline. A six-minute walk test (6MWT) or a cardiopulmonary exercise test (CPET) could not be performed due to ventilatory limitation.

**Figure 3:**
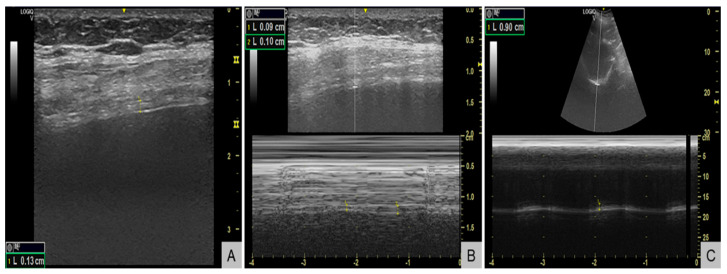
Diaphragm ultrasound demonstrating diaphragmatic weakness. (**A**) B-mode image showing reduced end-expiratory diaphragm thickness of 1.3 mm in the left hemidiaphragm (normal for women >1.7-2.0 mm). (**B**) M-mode image showing a reduced thickening fraction of 11% in the right hemidiaphragm (normal >20%). (**C**) M-mode tracing showing reduced inspiratory excursion of 0.9 cm (normal for women >1.5 cm during quiet breathing).

Respiratory evaluation also identified significant respiratory muscle weakness. Maximal inspiratory pressure (MIP) was −38 cmH_2_O (normal <−80 cmH_2_O in women), maximal expiratory pressure (MEP) +55 cmH_2_O (normal >100 cmH_2_O), and sniff nasal inspiratory pressure (SNIP) 34 cmH_2_O (normal >70 cmH_2_O), all below the lower limits of normal. Diaphragm ultrasound (Figure 3) revealed end-expiratory thickness 0.9 mm (right) and 1.3 mm (left) (normal for women >1.7–2.0 mm) (Figure 3A), thickening fraction 11% (right) (Figure 3B) and 16% (left) (normal >20%), and inspiratory excursion < 1.0 cm (Figure 3C) bilaterally (normal for women >1.5 cm during quiet breathing), consistent with diaphragmatic weakness. Polysomnography showed an apnea–hypopnea index of 13 events/hour, predominantly central hypopneas (n = 12), with a nadir oxygen saturation of 86% and no obstructive events. The patient’s body mass index was 23 kg/m^2^.

Because of the aforementioned findings, which were indicative of myopathy, electromyography was performed and demonstrated small, polyphasic motor unit potentials with early recruitment, indicative of a primary myopathic process. Antibodies against the acetylcholine receptor (AChR), muscle-specific kinase (MuSK), and low-density lipoprotein receptor-related protein 4 (LRP4) were negative. Genetic testing for Pompe disease was also negative. A muscle biopsy was proposed to further characterize the myopathy but was declined by the patient.

In parallel, because of clear PAH indications, right-heart catheterization was performed off non-invasive ventilation (NIV) on nasal cannula with FiO_2_ of 0.25, and confirmed precapillary pulmonary hypertension: right atrial pressure 8 mmHg (normal 2–6 mmHg), mean pulmonary artery pressure 42 mmHg (normal <20 mmHg), pulmonary arterial wedge pressure 8 mmHg (normal 6–12 mmHg), cardiac output 4.5 L/min (normal 4–8 L/min), cardiac index 2.6 L/min/m^2^ (normal 2.5–4.0 L/min/m^2^), and pulmonary vascular resistance 7.6 Wood units (normal <3 Wood units). Mixed venous oxygen saturation was 62% (normal >65%). Coronary angiography excluded obstructive coronary artery disease. Comprehensive autoimmune, vasculitis, amyloidosis, human immunodeficiency virus (HIV), hepatitis B (HBV) and C (HCV), and schistosomiasis panels were negative. Abdominal ultrasound and portal venous Dopplers excluded portal hypertension.

Since idiopathic PAH was strongly suspected, genetic testing was undertaken. However, because the coexisting myopathy was not fully compatible with IPAH alone, whole-genome sequencing was performed. This revealed a heterozygous pathogenic nonsense variant in the Glomulin (GLMN) gene (c.108C>A, p.Cys36*), classified as American College of Medical Genetics and Genomics (ACMG) class 5. No variants were identified in canonical PAH genes, including bone morphogenetic protein receptor type 2 (BMPR2). Dermatological evaluation, including dermoscopy, did not detect cutaneous or mucosal glomuvenous malformations. Family testing was declined.

The patient was treated with diuretics, long-term oxygen therapy, and nocturnal non-invasive ventilation. She was referred to a specialized pulmonary hypertension clinic, where combination therapy with ambrisentan and tadalafil was initiated in accordance with the 2022 ESC/ERS guidelines for patients with WHO-FC II–III IPAH and significant hemodynamic impairment [1]. No pulmonary oedema developed during vasodilator treatment, arguing against occult pulmonary veno-occlusive disease. On follow-up, she remained in WHO-FC II–III with persistent chronic hypercapnia and ventilatory muscle weakness. The diagnosis of idiopathic PAH with co-occurrence of a GLMN pathogenic variant and coexisting primary myopathy was retained.

## 3. Discussion

This case illustrates an unusual presentation of IPAH complicated by persistent hypercapnic respiratory failure due to primary myopathy, in which whole-genome sequencing revealed a pathogenic, previously described [4], GLMN variant. To our knowledge, this represents the first documented co-occurrence of IPAH and a GLMN mutation, raising the possibility of a novel association.

Pulmonary arterial hypertension is defined hemodynamically by precapillary pulmonary hypertension, mean pulmonary artery pressure (mPAP) > 20 mmHg, pulmonary arterial wedge pressure (PAWP) ≤ 15 mmHg, and pulmonary vascular resistance (PVR) > 2 Wood units, according to contemporary guidelines [1]. It belongs to Group 1 pulmonary hypertension and may occur as idiopathic, heritable, drug-induced, or associated with systemic conditions such as connective tissue disease, congenital heart disease, portal hypertension, HIV, and schistosomiasis [1,5]. Pathologically, PAH is characterized by pulmonary vascular remodeling, endothelial dysfunction, and abnormal smooth-muscle proliferation [6].

Idiopathic PAH accounts for the majority of “sporadic” cases once secondary causes are excluded [5]. The typical clinical profile includes progressive exertional dyspnea, right-sided heart failure, elevated NT-proBNP, and reduced exercise tolerance. Arterial blood gases usually reveal normocapnia or hypocapnia due to hyperventilation and increased dead space [2]. In contrast, our patient had chronic compensated hypercapnia (PaCO_2_ > 70 mmHg), which is distinctly unusual in IPAH. Hypercapnia in the setting of pulmonary hypertension should prompt evaluation for coexisting conditions, such as hypoventilation syndromes, chest wall abnormalities, or neuromuscular disease [7].

In this patient, the hypercapnia was explained by severe respiratory muscle weakness with abnormal diaphragm ultrasound and low maximal inspiratory/expiratory pressures. Electromyography confirmed a primary myopathic process, and the constellation of findings fulfilled established criteria for respiratory muscle weakness as outlined in American Thoracic Society/European Respiratory Society (ATS/ERS) standards [8]. Importantly, obesity hypoventilation syndrome, obstructive sleep apnea, and chronic obstructive or interstitial lung disease were excluded. Polysomnography demonstrated central but not obstructive events, and body mass index was normal. Thus, hypercapnia reflected ventilatory pump failure due to myopathy, coexisting with IPAH rather than being a consequence of PAH itself.

The diagnosis of IPAH in this patient was robust, as systematic evaluation excluded other causes of pulmonary hypertension [1]. Left-heart disease was ruled out by normal wedge pressure and preserved left ventricular function. Chronic thromboembolic pulmonary hypertension was excluded by a normal ventilation/perfusion scan. Group 3 PH was unlikely, with no evidence of obstructive spirometry, parenchymal lung disease, or obstructive sleep apnea. The patient’s mild hypoventilation reflected underlying respiratory muscle weakness, but this was insufficient to explain the hemodynamic profile of precapillary pulmonary hypertension. Pulmonary veno-occlusive disease was unlikely given HRCT findings, the absence of interlobular septal thickening or lymphadenopathy, and the absence of pulmonary oedema after vasodilator therapy [9]. A broad serological screen excluded connective tissue disease, vasculitis, HIV, schistosomiasis, and hepatitis B & C. Thus, idiopathic PAH remained the most appropriate classification.

Genetic predisposition is increasingly recognized as central in IPAH pathogenesis. Mutations in BMPR2 account for most heritable cases, while variants in KCNK3, TBX4, SOX17, CAV1, ATP13A3, and others have been described [3,10,11]. Penetrance is incomplete, but carriers often exhibit earlier onset and more severe disease [10,11]. For this reason, current guidelines recommend genetic testing in idiopathic and familial PAH [1,3]. Our patient had no pathogenic variants in canonical PAH genes, but whole-genome sequencing identified a heterozygous nonsense variant in GLMN (c.108C>A, p.Cys36*), classified as ACMG class 5. Notably, the nonsense variant identified in our patient was among the pathogenic GLMN mutations originally reported in patients with GVMs [4]. The absence of cutaneous or mucosal lesions in our case is therefore consistent with the incomplete penetrance and variable expressivity described in GLMN-related disease.

GLMN encodes glomulin, a protein crucial for vascular smooth-muscle cell and pericyte maturation [4]. Pathogenic variants cause glomuvenous malformations (GVMs), which typically manifest as cutaneous or mucosal venous lesions. Penetrance is incomplete, and some carriers remain unaffected [12]. A “two-hit” model has been proposed, whereby a germline mutation and a somatic second hit are required for lesion formation [12,13]. Our patient had no cutaneous or mucosal lesions, consistent with incomplete penetrance. To date, no evidence directly implicates GLMN in pulmonary vascular disease.

While GLMN has not been previously linked to pulmonary vascular disease, its role in vascular smooth-muscle biology provides a plausible mechanistic link, although causality cannot be inferred, and the possibility of coincident, unrelated conditions should be acknowledged. Loss of glomulin function may impair vascular smooth-muscle maturation and vessel wall stability, lowering the threshold for maladaptive pulmonary arteriolar remodeling under hemodynamic stressors such as hypoxia, shear stress, or inflammation [4,6,14]. The absence of systemic venous lesions suggests tissue-specific expressivity, with the pulmonary vascular bed being uniquely susceptible. These findings support an association and mechanistic plausibility, but not causality. The coexistence of idiopathic PAH with an unrelated pathogenic GLMN variant remains possible, highlighting the need for cautious interpretation.

This case carries several clinical implications. First, hypercapnia in suspected IPAH should prompt formal evaluation of respiratory muscle function, including maximal inspiratory/expiratory pressures and diaphragm ultrasound, to avoid misclassification as Group 3 PH. Second, extended genetic testing beyond canonical PAH panels may identify novel variants with potential relevance to pulmonary vascular biology, even if pathogenicity cannot yet be established. Third, documenting such rare variants is crucial to inform future registries and translational studies, which may reveal new molecular pathways in PAH.

Limitations must be acknowledged. This is a single case, and causality cannot be inferred. Muscle biopsy and family genetic testing were proposed but declined. Tissue-specific somatic second hits cannot be excluded. Nevertheless, the convergence of IPAH, primary myopathy with diaphragmatic weakness, and a pathogenic GLMN variant highlights a previously unrecognized intersection of vascular and neuromuscular biology.

## 4. Conclusions

This study presents the first documented case of IPAH in a patient carrying a pathogenic GLMN variant, complicated by chronic hypercapnic respiratory failure due to primary myopathy. Hypercapnia is atypical in IPAH and, in this instance, was explained by diaphragmatic weakness, highlighting the importance of respiratory muscle evaluation in such patients. Although causality between GLMN and pulmonary vascular disease cannot be established, the recognized role of glomulin in vascular smooth-muscle development provides mechanistic plausibility. This case provides a hypothesis-generating signal, highlighting a potential novel association between IPAH and GLMN, and broadens the genetic spectrum associated with IPAH, warranting further investigation into pulmonary vascular biology and identifying GLMN as a potential candidate for further research.

## Data Availability

The original contributions presented in this study are included in the article. Further inquiries can be directed to the corresponding author.

## Abbreviations

The following abbreviations are used in this manuscript:

6MWT	six-minute walk test
ABG	arterial blood gases
AChR	antibodies against the acetylcholine receptor
ACMG	American College of Medical Genetics and Genomics
ATS	American Thoracic Society
BMPR2	bone morphogenetic protein receptor type 2
CPET	cardiopulmonary exercise test
DLCO	diffusing capacity for carbon monoxide
ESC/ERS	European Society of Cardiology/European Respiratory Society
FEV_1_	forced expiratory volume in one second
FiO_2_	inspired oxygen fraction
FVC	forced vital capacity
GLMN	glomulin
GVMs	glomuvenous malformations
HBV	hepatitis B
HCO_3_^−^	bicarbonate
HCV	hepatitis C
HIV	human immunodeficiency virus
HRCT	high-resolution computed tomography
IPAH	idiopathic pulmonary arterial hypertension
LRP4	low-density lipoprotein receptor-related protein 4
MEP	maximal expiratory pressure
MIP	maximal inspiratory pressure
mPAP	mean pulmonary artery pressure
MuSK	muscle-specific kinase
NIV	non-invasive ventilation
NT-proBNP	N-terminal pro–B-type natriuretic peptide
PaCO_2_	partial pressure of carbon dioxide
PAH	pulmonary arterial hypertension
PaO_2_	partial pressure of oxygen
PAWP	pulmonary arterial wedge pressure
PH	pulmonary hypertension
PVR	pulmonary vascular resistance
SNIP	sniff nasal inspiratory pressure
TLC	total lung capacity
WHO-FC	World Health Organization functional class
